# Characterization and Mapping of a Rolling Leaf Mutant Allele *rlT73* on Chromosome 1BL of Wheat

**DOI:** 10.3390/ijms25074103

**Published:** 2024-04-07

**Authors:** Lin Huang, Meijuan Gan, Wenzhuo Zhao, Yanling Hu, Lilin Du, Yuqin Li, Kanghui Zeng, Dandan Wu, Ming Hao, Shunzong Ning, Zhongwei Yuan, Lihua Feng, Lianquan Zhang, Bihua Wu, Dengcai Liu

**Affiliations:** 1 State Key Laboratory of Crop Gene Exploration and Utilization in Southwest China, Sichuan Agricultural University, Chengdu 611130, China; 2 Triticeae Research Institute, Sichuan Agricultural University, Chengdu 611130, China

**Keywords:** wheat, rolling leaf, miRNA, HD zipper class III transcription factor

## Abstract

Leaf rolling is regarded as an important morphological trait in wheat breeding. Moderate leaf rolling is helpful to keep leaves upright and improve the photosynthesis of plants, leading to increased yield. However, studies on the identification of genomic regions/genes associated with rolling leaf have been reported less frequently in wheat. In this study, a rolling leaf mutant, T73, which has paired spikelets, dwarfism, and delayed heading traits, was obtained from a common wheat landrace through ethyl methanesulfonate mutagenesis. The *rlT73* mutation caused an increase in the number of epidermal cells on the abaxial side and the shrinkage of bulliform cells on the adaxial side, leading to an adaxially rolling leaf phenotype. Genetic analysis showed that the rolling leaf phenotype was controlled by a single recessive gene. Further Wheat55K single nucleotide polymorphism array-based bulked segregant analysis and molecular marker mapping delimited *rlT73* to a physical interval of 300.29–318.33 Mb on the chromosome arm 1BL in the Chinese Spring genome. We show that a point mutation at the miRNA165/166 binding site of the HD zipper class III transcription factor on 1BL altered its transcriptional level, which may be responsible for the rolling leaf phenotype. Our results suggest the important role of *rlT73* in regulating wheat leaf development and the potential of miRNA-based gene regulation for crop trait improvement.

## 1. Introduction

The improvement of yield is one of the main objectives of crop breeding. Leaves are the main organs of photosynthesis in plants [1]. Leaf rolling is a complex agronomic trait that is affected by both genotype and environment. Appropriate leaf rolling is helpful to maintain the erectness of leaves and improve light acceptance, which leads to increased yield [2,3]. Therefore, the study of leaf morphology is considered an important part of basic theory and practical breeding applications.

Leaf rolling is a complex trait that is controlled by a single gene with a major effect [1,2,3,4] or multiple genes with minor effects [5,6,7]. In field crops, plenty of genes and their underlying pathways involved in regulating leaf rolling have been extensively studied. In rice, more than 70 genes/quantitative trait loci (QTLs) for the leaf rolling trait have been mapped, and at least 17 mutants with rolled leaves have been characterized [8]. Some genes modulate rolling leaf phenotypes by regulating the morphology, number, size, or distribution of the bulliform cells [1]. For example, knock-out of *Rice outermost cell-specific gene 5* (*Roc5*) induced the abaxial leaf rolling phenotype because of the increased number and size of bulliform cells, while overexpression of *Roc5* led to an adaxial rolling leaf [4]. Overexpression of *OsZHD1* led to an increased number and the abnormal arrangement of bulliform cells, causing abaxial rolling leaf [9]. In addition, genes regulating mesophyll cells, sclerenchyma cells, epidermal cells, and cuticular wax also have important roles in determining leaf rolling, such as *SLL1* (*SHALLOT-LIKE1*) [10], *CFL1* (*CURLY FLAG LEAF1*) [11], and *NRL2* (*NARROW AND ROLLED LEAF2*) [12]. In maize, six rolling leaf mutants have been reported, including *leafbladeless1* (*lbl1*) [13], *rolled leaf1* (*rld1*) [14], *roll-leaf-above-ear* (*rlae*) [15], mutant in maize hybrid Sunuo5670 [16], *swl* [17], and *abaxial rolling leaf1* (*abrl1*) [6]. Among them, *Lbl1*, encodes a SUPPRESSOR OF GENE SILENCING 3 homologue protein that is required for specifying adaxial cell identity in leaves and leads to abaxialized leaves [13]. *Rld1* is a class III homeodomain-leucine zipper (HD-ZIP III) protein, and the mutation in the miRNA166 target sequence of *Rld1* shows a rolling leaf phenotype [18].

In wheat, studies regarding leaf rolling and identification of genomic regions/genes are less reported. A study on the RIL (recombinant inbred line) population derived from the cross between varieties NI5439 and HD2012 identified 12 QTLs associated with flag leaf rolling under moisture stress conditions [5]. In all field experiments, two consistent QTLs were identified for the rolled leaf trait on chromosomes 1A (*QRl.hwwg-1AS*) and 5A (*QRl.hwwg-5AL*) of JagMut1095 [19]. In addition, stable QTLs associated with leaf rolling were also reported in wheat relatives such as rye (*Secale cereale*) [20] and tetraploid wheat [7]. Recently, studies have shown that the *Abnormal Plant Architecture-1* (*APA1*) locus encodes a class III homeodomain-leucine zipper (HD-ZIP III) transcription factor (*HB-D2*) that is associated with multiple morphological changes, including upward-curled leaves [21]. The *TaMYB5* transcription factor regulates leaf rolling by directly binding to the promoter through the AC cis-acting element to induce the expression of *TaNRL1* [3].

In this study, we identified a wheat rolling leaf mutant, T73, from an ethylanesulfonate (EMS)-mutagenized population of the common wheat landrace ‘Chinese Spring’ (CS hereafter). Morphological characterization, microscopic observation, genetic analysis, and subsequent gene mapping, as well as RNA-sequencing, were performed to search for the candidate genes responsible for the rolling leaf phenotype. This research provides the foundation for further cloning of the casual gene *rlT73* and a better understanding of the regulation of leaf development in wheat.

## 2. Results

### 2.1. Phenotype Characterization of Mutant Line T73

**Figure 1:**
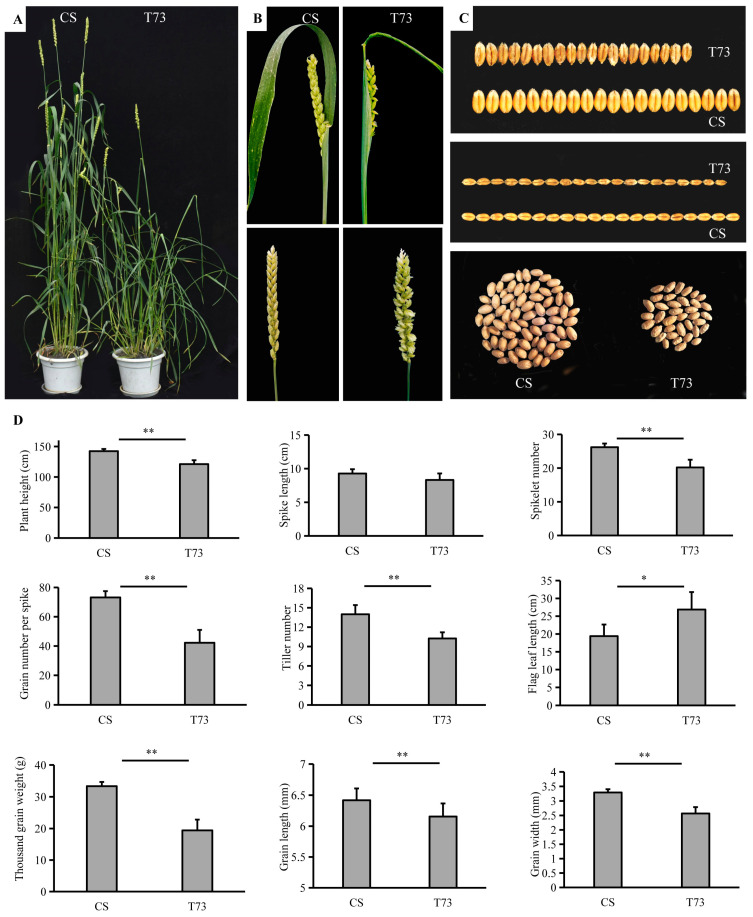
Phenotypic characterization of T73 mutant plants. (**A**) CS and T73 plants grown in a field environment. (**B**) Spike morphology of CS and T73. (**C**) Seeds of CS and T73. (**D**) Statistics of plant height, spike length, spikelet number, grain number per spike, tiller number, flag leaf length, thousand grain weight, grain length, and grain width of CS and T73. The values for grain length and grain width were an average of 50 grains. * *p* < 0.05; ** *p* < 0.01.

**Figure 2:**
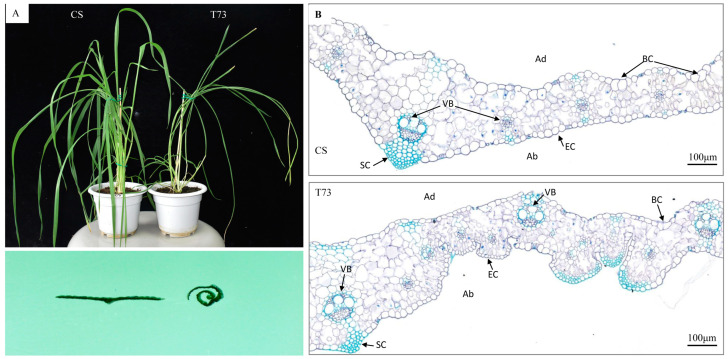
Phenotype changes of CS and T73 leaves. (**A**) Plant stature (**top**) and transverse leaf sections (**bottom**) of CS and T73. (**B**) Toluidine blue-stained cross sections of CS and T73 leaves. BC, bulliform cell; VB, vascular bundles; SC, sclerenchyma cells; EC, epidermal cell; Ad, adaxial; and Ab, abaxial.

The EMS-derived mutant T73 started to show adaxial leaf rolling when the fourth leaf emerged under growth chamber conditions. The typical rolling leaf phenotype became increasingly evident during plant growth. Beginning from the tillering stage, the leaf blades of the T73 displayed a severe rolling or even shallot-like rolling phenotype, whereas the corresponding wild-type leaves were almost flat (Figure 1A and Figure 2A). At the mature stage, the T73 exhibits paired spikelets in comparison with the CS, which has one spikelet per rachis node (Figure 1B). In addition, major agronomic traits of T73 were affected (Figure 1C,D). For example, the plant height, spikelet number, tiller number, grain number per spike, and thousand grain weight were significantly lower than those of the wild-type, while the flag leaf was significantly longer compared with that of the wild-type. There is no significant difference in spike length between the CS and T73 plants. The rolling leaf phenotype is a prominent feature of the T73 plants throughout the growth stage; therefore, we mainly focused on this phenotype for subsequent analysis.

Paraffin-cross sectioning of leaves at the elongation stage showed that the bulliform cells in the T73 mutant were deflated and even not obvious. Although the size of epidermal cells on the abaxial side was reduced, the number of epidermal cells was largely increased in T73 compared with those in the wild-type. Therefore, the increase in the number of epidermal cells on the abaxial side and the deflated bulliform cells on the adaxial side of the leaf blade are the major morphological changes observed in the leaf blade of T73.

### 2.2. Inheritance of the Rolling Leaf Phenotype

**Figure 3:**
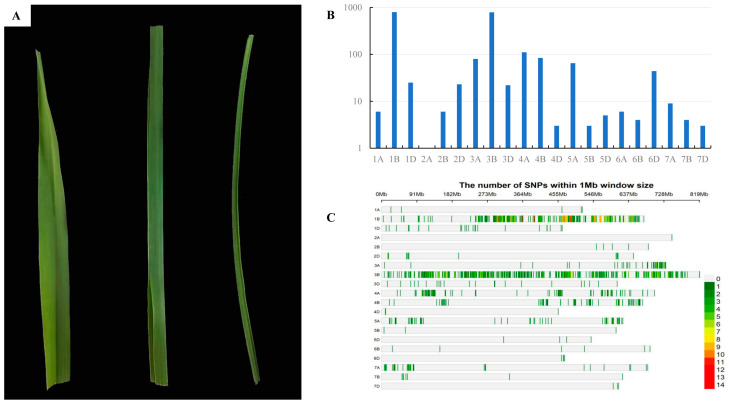
Genetic characterization of *rlT73*. (**A**) Leaves of CS (left), F_1_ of T73 × CS (middle), and T73 (right). (**B**) Number of SNPs distributed on wheat chromosomes in the T73 × AK58 mapping population. (**C**) The enrichment of SNPs on wheat chromosomes in the T73 × AK58 mapping population.

All F_1_ plants displayed an intermediate phenotype (Figure 3A). Among the 200 F_2_ individuals and their corresponding F_2:3_ families, 46 families showed the homozygous rolling leaf phenotype, 101 were segregating, and 53 were homozygous for the WT phenotype, which fits the segregation ratio of 1:2:1 (χ_1:2:1_^2^ = 0.259, *p* = 0.879), indicating that the rolling leaf phenotype of T73 was conferred by a single recessive genetic locus tentatively designated as *rlT73*.

### 2.3. Molecular Mapping of rlT73

Genotyping of wild-type bulk (W-bulk) and mutant bulk (M-bulk) of a T73 × AK58 segregating population using a wheat55K SNP array identified the highest number of polymorphic SNPs (794) on chromosome 1B, accounting for 38.1% of all screened polymorphic SNPs (Figure 3B), indicating that the target gene was most likely on chromosome 1B. The SNPs on 1B were mainly enriched in a region from 237 to 551 Mb (Figure 3C) in the Chinese Spring reference genome (RefSeq v1.1), suggesting that the *rlT73* is probably located in this interval.

**Figure 4:**
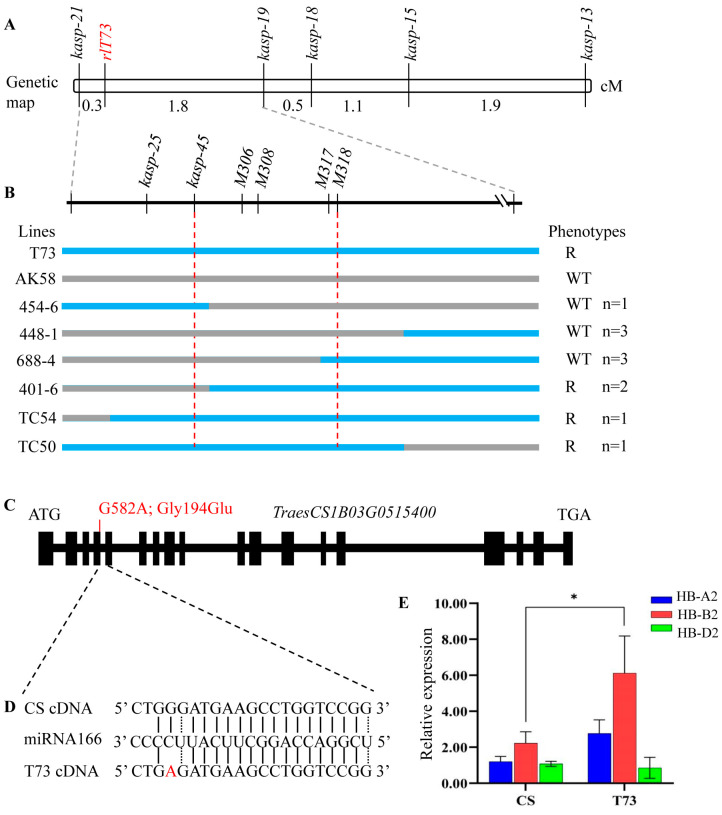
Molecular mapping and candidate gene analysis of *rlT73*. (**A**) Genetic map of *rlT73.* (**B**) the molecular markers used to narrow down the genetic interval of *rlT73*. 45-6, 448-1, 688-4, 401-6, TC50, and TC54 are homozygous lines that displayed recombinant events in the *rlT73* region. The actual number (n) of each recombinant event is listed. R, rolling leaf phenotype; WT, wild-type leaf. (**C**) Gene structure of *TraesCS1B03G0515400*. (**D**) Schematic representation of the missense variant involved in the miRNA166 binding site of *TraesCS1B03G0515400*. (**E**) Expression levels of *TraesCS1B03G0515400* (*HB-B2*) and its homeologs (*HB-A2* and *HB-D2*) in wheat leaves of CS and T73. Plants were grown in growth chambers at 22 °C with an 18 h light/6 h dark photoperiod. Leaves were collected at the jointing stage. * *p* < 0.05.

Five KASP markers surrounding the interval of *rlT73* were designed and used to genotype the 193 F_2_ plants of T73 × AK58. Linkage analysis indicated that *rlT73* was mapped between markers *kasp-19* and *kasp-21* (Figure 4A), corresponding to the physical region of 275–370 Mb (CS RefSeq v2.1). The order and position of markers are consistent in both the linkage map and the physical map. To define the better position of *rlT73*, six new molecular markers, including one sanger sequencing marker, two KASP markers, and three Indel markers based on SNPs and InDels between the CS and AK58 genome sequences, were further developed and used to genotype another 663 F_2_ individuals. The *rlT73* candidate region was delimited to a ~18 Mb physical interval of 300.29–318.33 Mb, flanked by markers *kasp-45* and *M318* on chromosome arm 1BL (Figure 4B).

### 2.4. Analyzing Candidate Genes Based on RNA-Seq Data

A BLAST search of the flanking marker sequences on the CS reference genome version 2.1 revealed 72 high-confidence annotated genes in the *rlT73* localization interval (Table S1). Some of these genes, such as the cellulose synthase gene (*TraesCS1B03G0493300*), receptor protein kinase genes (*TraesCS1B03G0493600* and *TraesCS1B03G0493700*), MYB transcription factor family gene (*TraesCS1B03G0509200*), and homeobox leucine-zipper gene (*TraesCS1B03G0515400*), are candidate genes of particular interest.

RNA-seq analysis of mutant bulk (M-bulk-2) and T73 revealed that *TraesCS1B03G0515400* was the only expressed candidate gene in the *rlT73* interval. *TraesCS1B03G0515400* displayed a G>A mutation at position 581 base pair (bp) of the coding sequence (Gly194Glu) (Figure 4C and Figure S1). *TraesCS1B03G0515400* spans 8532 bp from the start to the stop codon, with a coding sequence of 2523 bp. It encodes a class III homeodomain-leucine zipper (HD-ZIP III) transcription factor protein (designated as *HB-B2*) that is homologous to copies of *TraesCS1A02G157500* (*HB-A2*) and *TraesCS1D02G155200* (*HB-D2*) on the A and D subgenomes. The EMS-derived mutation was positioned at the miR165/166 complementary site of *HB-B2* (Figure 4D). Quantitative PCR revealed that *HB-B2* was expressed significantly higher (*p* < 0.01) in the mutant plants than wild-type plants, while the transcripts of its homologs *HB-A2* and *HB-D2* were not altered significantly (Figure 4E). These results demonstrated that *HB-B2* could be the causal gene responsible for the mutant phenotypes.

## 3. Discussion

Specific leaf morphologies and ideal plant architecture contribute largely to the yield potential of crops. Leaf rolling is a complex and agronomically important trait that is controlled by a single gene with a major effect or multiple genes with minor effects [4,6,22,23]. Peleg et al. [7] reported 14 rolling leaf QTLs on chromosomes 1A, 2A, 2B, 4B, 5A, 5B, 6A, 6B, 7A, and 7B of *T. turgidum* ssp. *dicoccoides*. Bian et al. [19] detected two consistent QTLs for rolling leaf on chromosomes 1A (*QRl.hwwg-1AS*) and 5A (*QRl.hwwg-5AL*) in a wheat EMS mutant from Jagger. Verma et al. [5] found 12 rolling leaf QTLs on chromosomes 1B, 2A, 2B, 2D, 3A, 4A, 4B, 5D, and 6BL under moisture stress conditions in wheat. Among them, *Qlr.nhv-1B* was delimited to a 3.2 Mb interval in Chinese Spring reference genome sequence v2.1 between the markers *gwm153* (635,904,863 bp) and *AX-95149749* (639,067,972 bp). In the present study, the *rlT73* was mapped to the interval between 300.29 and 318.33 Mb on 1BL, which is different from the *Qlr.nhv-1B*. Therefore, the *rlT73* is most likely a new locus for rolling leaf.

The rolling leaf phenotype can be caused by changes in the structure of a particular cell type or multiple cell types [24]. Bulliform cells located on the upper epidermis of leaves play a critical role in regulating leaf rolling in grass species [2]. The rice mutant *crm1-D* with an inward-curve leaf phenotype was associated with the size reduction of bulliform cells [25]. A previous study showed that morphological changes of the bulliform cells at the adaxial side of the leaf blade and the abnormality of sclerenchymatous cells at the abaxial side are two main factors contributing to the rolling leaf phenotype of rice miR166 knockdown lines [26]. Using anatomical analyses, we showed that the increase in the number of epidermal cells at the abaxial side and the shrinkage of bulliform cells at the adaxial side of the leaf blade may be responsible for the rolling leaf phenotype of T73 plants.

The *rlT73* candidate region was delimited to a ~18 Mb physical interval on 1BL, including 72 high-confidence annotated genes. Based on RNA-seq analyses, *TraesCS1B03G0515400* encodes an HD-ZIP transcription factor (*HB-B2*) and is regarded as the best candidate for *rlT73*. HD-ZIP-type proteins have been reported to be associated with the regulation of inflorescence, leaf, and stem development in plants [27,28]. A previous study has reported that overexpression of the mutated resistant version of *HB-D2* in wheat displayed severe leaf rolling and extreme dwarfism [21]. Further fine mapping and functional validation of the candidate genes of *rlT73* are necessary.

Previous studies demonstrated that mutations in the miRNA165/166 complementary site of homeologs *HB-A2* and *HB-D2* in wheat caused upward-curled leaves, paired spikelets, and reduced plant height [21,28]. In this study, we found a single nucleotide substitution (G581A; Gly194Glu) at the miRNA165/166 binding site of *HB-B2* altered its transcriptional level, which may be responsible for the rolling leaf phenotype. These results suggest that the *HB-2* homeologs may have a redundant role in regulating leaf development in wheat. Homoeologs genes such as *GPC-A1* and *GPC-D1* have overlapping functions in the regulation of monocarpic senescence and nutrient remobilization in wheat [29].

A previous study showed that the ‘Cadenza’ mutant line carrying a single nucleotide change, C589T (Pro197Ser), in the central part of the miRNA165/166 binding site of *HB-B2* had weaker phenotypic effects with needle-like secondary spikelets [21]. In the present study, we found that the *rlT73* mutant carrying mutation (G581A; Gly194Glu) in the 3’ end of miRNA165/166 of *HB-B2* showed severe rolling leaf phenotype, paired spikelets comparable to those of *rHb-D2* mutants [21,28]. Zhang et al. [30] identified several mutation sites on the wheat domestication gene *Q* and showed that mutations in different domains resulted in distinct phenotypes. Moreover, point mutations interfere with the miRNA172-directed cleavage of *Q* transcripts, which contributes to reduced height/compact spike phenotypes [31] and improved processing quality [32]. These results point to an emerging theme of breeding, where mutations in miRNA binding sites disrupting the cleavage of the developmental gene transcripts could be a strategy for crop trait improvement. Although *rlT73* displayed negative effects on major agronomic traits, which precludes its direct use in breeding, the cloning of *rlT73* and its targets may help to understand the molecular basis of leaf development and to develop wheat plants with ideal plant architecture.

## 4. Materials and Methods

### 4.1. Plant Materials and EMS Mutagenesis

The wild-type (WT) parent common wheat Sichuan landrace CS was treated with 0.5% EMS to construct a mutant population in 2017. Over three thousand EMS-treated CS seeds were sown in fields to generate M_1_ plants in November 2017 in Chongzhou, Sichuan, China. A total of 1132 individual plants were harvested separately and planted in November 2018 in Chongzhou in a 2 m row, with 30 cm between rows. We identified an M_2_ mutant (T73) showing adaxially rolled-leaves, paired spikelets, dwarfism, and delayed heading under field conditions. The mutant plant was further used for two generations of self-pollination in a growth chamber. M_4_-generation plants displaying a stable rolling leaf phenotype were used for subsequent analysis.

The mutant T73 was crossed with the wild-type CS to construct the F_1_, F_2_, and F_2:3_ populations (T73 × CS) for genetic analysis of the rolling leaf phenotype, and it was further crossed with the common wheat cultivar AK58 to construct the mapping population (T73 × AK58). Part of the F_2:3_ families were grown in the experimental field of Sichuan Agricultural University, Wenjiang, Sichuan Province, China.

### 4.2. Morphological Traits and Microscopic Observations

Five morphological traits of T73 and CS were measured at physiological maturity. The plant height, flag leaf length, spike length, spikelet number, and grain number per spike were recorded in the first culm and spike of each plant. In addition, tiller number and thousand grain weight were also recorded, as described by Gong et al. [33]. The mean measurement of at least five individuals was estimated for each trait.

To investigate the formation mechanism of rolled leaves in T73, paraffin cross-sectioning of leaves was conducted at the elongation stage. The leaf samples were firstly fixed with FAA (formaldehyde-acetic acid-ethanol) fixative, dewaxed with water, stained with toluidine blue (toluidine blue staining solution, Servicebio, Wuhan, China), transparently mounted, and subjected to optical microscope examination and image acquisition [34] according to the manual of Wuhan Servicebio Technology Co., Ltd.

### 4.3. Genotyping

To map the potential loci modulating the rolled-leaf phenotype, 20 wild-type and 20 mutant individuals from the F_2_ population of T73 × AK58 were collected to construct W-bulk and M-bulk, respectively. The total genome DNA of the parents and bulks was extracted using a plant genomic DNA kit (Tiangen Biotech, Beijing, China). DNA purity and integrity were assessed and confirmed. Chip-based genotyping was carried out using the Wheat55K SNP array containing 53,063 markers by China Golden Marker (Beijing) Biotech Co. Ltd. (Beijing, China). The monomorphic, heterozygous, and low-quality SNPs with ambiguous SNP signals were filtered out. Markers showed homozygous genotypes among T73, AK57, and M-bulk were retained, and a graph of the enrichment of SNPs on the whole genome was plotted using a sliding widow of 1 Mb.

### 4.4. Molecular Marker Development

SNPs linked to the rolling leaf phenotype were identified by Wheat55K SNP analysis and were chosen to design the competitive allele-specific PCR (KASP) markers. In addition, we aligned the CS and AK58 genomic sequences to identify insertion/Deletions (InDels) within the target region for InDel marker development. All primers were firstly tested for polymorphisms on the parent lines, W-bulk, and mutant bulk. Polymorphic markers that could be reliably scored were used to genotype the F_2_ segregation population to construct a genetic linkage map (Table S2).

### 4.5. Candidate Gene Analysis

The sequences of flanked markers were aligned to the Chinese Spring reference genome sequence v2.1 to obtain the physical positions. Gene annotations between the flanking markers of the CS genome were retrieved from the online databases (http://202.194.139.32/; accessed on 9 October 2023). An equal quantity of leaves from 20 mutant plants in the F_2_ population of T73 × CS were chosen to construct a M-bulk-2. RNA extraction, library construction, and sequencing of M-bulk-2 and T73 mutants were carried out by Frasergen Bioinformatics Co., Ltd. (Wuhan, China). Raw RNA-seq reads were trimmed using Trimmomatic software v0.32 to remove low-quality reads and adaptors [35]. To facilitate candidate gene mining, the clean RNA-Seq reads of M-bulk-2 and T73 were aligned to the Chinese Spring reference genome sequence using HISAT 2.0 (V 2.2.1) with default parameters. The alignment file in BAM format was indexed using Samtools software version 1.9 and displayed using Integrative Genomics Viewer (IGV) to manually identify EMS-derived polymorphic sites in the candidate region.

### 4.6. Quantitative Real-Time PCR (qRT-PCR) Analysis

Leaf RNA was isolated using TRIzol reagent (Tiangen, Beijing, China), and the first-strand complementary DNA (cDNA) was reverse transcribed from 1 μg of total RNA using the TaKaRa PrimeScriptTMRT Reagent Kit (Takara, Dalian, China). The qRT-PCR reactions were conducted on a Bio-Rad CFX96 Real-Time PCR detection system (Bio-Rad, Hercules, CA, USA) with SYBR qPCR Master Mix (Q711, Vazyme, Nanjing, China). Transcript levels were calculated using the 2^−ΔΔCT^ method [36]. *TaGAPDH* was used as the endogenous control for normalizing gene expression.

## Data Availability

The data presented in this study are available in the Supplementary Materials.

## Supplementary Materials

The supporting information can be downloaded at: https://www.mdpi.com/article/10.3390/ijms25074103/s1.
