# Supplementary material for: Characterization and Mapping of a Rolling Leaf Mutant Allele rlT73 on Chromosome 1BL of Wheat
Source: Int J Mol Sci. 2024 Apr 7;25(7):4103. doi: 10.3390/ijms25074103 (PMC11012251; doi:10.3390/ijms25074103)
Supplement: Supplementary file 1 [file ijms-25-04103-s001.zip › Figure S1.pdf]

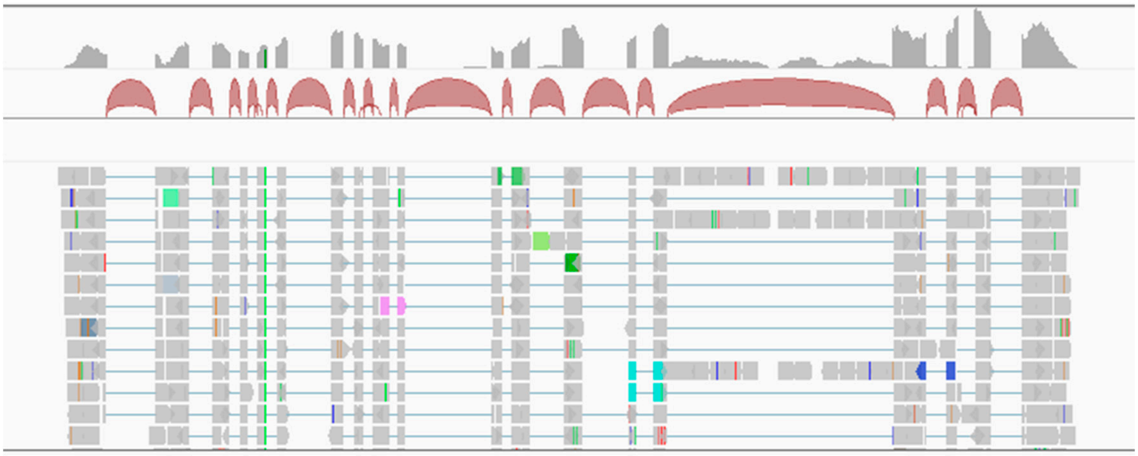

Figure S1 Mutation sites in *TraesCS1B03G0515400* visualised in Integrated Genomics Viewer (IGV). Consistent point mutation in *HB-B2* is marked in green.
